# Skull’s Photoacoustic Attenuation and Dispersion Modeling with Deterministic Ray-Tracing: Towards Real-Time Aberration Correction

**DOI:** 10.3390/s19020345

**Published:** 2019-01-16

**Authors:** Leila Mohammadi, Hamid Behnam, Jahan Tavakkoli, Mohammad R. N. Avanaki

**Affiliations:** 1 Department of Biomedical Engineering, Islamic Azad University, Science and Research Branch, Tehran 1477893855, Iran; lemoelec@gmail.com; 2 Department of Biomedical Engineering, Iran University of Science and Technology, Tehran 1684613114, Iran; 3 Department of Physics, Ryerson University, Toronto, ON M5B 2K3, Canada; jtavakkoli@ryerson.ca; 4 Institute for Biomedical Engineering, Science and Technology (iBEST), Keenan Research Center for Biomedical Science, St. Michael’s Hospital, Toronto, ON M5B 1W8, Canada; 5 Department of Biomedical Engineering, Wayne State University, Detroit, MI 48202, USA; ft5257@wayne.edu; 6 Department of Dermatology, Wayne State University School of Medicine, Detroit, MI 48201, USA; 7 Barbara Ann Karmanos Cancer Institute, Detroit, MI 48201, USA

**Keywords:** acoustic attenuation, acoustic dispersion, mode conversion, numerical simulation, photoacoustic imaging, skull phantom, transcranial brain imaging

## Abstract

Although transcranial photoacoustic imaging has been previously investigated by several groups, there are many unknowns about the distorting effects of the skull due to the impedance mismatch between the skull and underlying layers. The current computational methods based on finite-element modeling are slow, especially in the cases where fine grids are defined for a large 3-D volume. We develop a very fast modeling/simulation framework based on deterministic ray-tracing. The framework considers a multilayer model of the medium, taking into account the frequency-dependent attenuation and dispersion effects that occur in wave reflection, refraction, and mode conversion at the skull surface. The speed of the proposed framework is evaluated. We validate the accuracy of the framework using numerical phantoms and compare its results to k-Wave simulation results. Analytical validation is also performed based on the longitudinal and shear wave transmission coefficients. We then simulated, using our method, the major skull-distorting effects including amplitude attenuation, time-domain signal broadening, and time shift, and confirmed the findings by comparing them to several ex vivo experimental results. It is expected that the proposed method speeds up modeling and quantification of skull tissue and allows the development of transcranial photoacoustic brain imaging.

## 1. Introduction

Photoacoustic imaging (PAI) is a non-ionizing imaging modality, based on the photoacoustic (PA) effect. PAI combines the high absorption contrast of optical imaging with the high spatial resolution of ultrasound imaging to visualize tissue chromophores in the optical quasi-diffusive or diffusive regime. In PAI, the biological tissue is illuminated with a short-pulsed laser beam that results in the generation of internal acoustic signals via the thermoacoustic effect. The subsequent ultrasound signal propagating from within the tissue is then detected by several wide-band ultrasonic transducers located outside the tissue. The ultrasound signal is then used to form an image through a reconstruction algorithm [1,2]. Several preclinical studies have demonstrated the tremendous utility of PAI in providing functional, molecular, and structural information about biological tissues [3,4,5,6,7,8,9].

Due to the functional imaging capability of PAI, one of the fast-emerging applications for this technology has been transcranial brain imaging in small and large animals [6,10,11,12,13,14,15,16,17,18,19,20]. Measuring cerebral hemodynamic parameters using PAI is arguably one of the most desired applications in brain functional imaging, tumor hypoxia evaluation, and even cancer therapy [10,21]. In recent works different types of photoacoustic computed tomography systems have been used for non-invasive, high resolution, and deep functional mouse brain imaging with the skull intact to study brain hemodynamics [22,23,24,25].

Despite promising results obtained from small animal, i.e., mice, PA brain imaging experiments, the signal deteriorating effect of skull is significant and has affected the image quality [26,27,28]. This issue has also been expressed at earlier works in transcranial ultrasound wave propagation in the context of ultrasound imaging or therapy, which demonstrating the unwanted distortions of the skull in the location and shape of the therapeutic focus, along with a reduction in focal intensity [29,30,31,32,33,34,35]. Pinton et al. quantified numerically and experimentally the attenuation, scattering, and thermal absorption of ultrasound in the human skull bone [36].

Skull-induced deterioration of the PA signal, so called “aberrations”, is due to attenuation, dispersion, and longitudinal to shear mode conversion [27,37,38,39]. Skull bone represents a highly acoustical impedance mismatch and dispersive barrier for the propagation of the ultrasound/PA wave [40]. Acoustic attenuation is due to absorption and scattering of the skull tissue or the reflection at the skull–tissue interfaces [38]. Attenuation is a frequency-dependent phenomenon and affects the amplitude of the signal [37,41]. Acoustic dispersion is the dependency of the sound speed or phase velocity to the frequency. It distorts the phase information in the PA signal [37]. Several groups investigated the effects of acoustic attenuation on PA signals and its relation to dispersion [42,43,44,45]. It has been shown that the frequency-dependent reduction of amplitude and corresponding dispersion of the acoustic waves contributes to the broadening of the PA signals and reduces the resolution of the reconstructed images [42]. Mode conversion between longitudinal and transverse waves occurs when a wave encounters an interface between materials of different acoustic impedances with the incident angle not being normal to the interface [39]. The combination of these skull-induced aberrations diminishes the quality of the PA images [46,47].

There have been several studies that modeled skull aberrations in transcranial ultrasound and PA imaging based on ray-acoustic model [35], angular spectrum method [31,39,48,49], and full-wave propagation equation [32,33,34,50]. The most common numerical methods to model the skull are finite-difference, finite-element, and boundary-element [32,33,34,36,44,50,51,52,53,54]. K-Wave, is a MATLAB toolbox, uses a finite-difference time-domain (FDTD) algorithm for modeling. k-space pseudo-spectral algorithm is the faster version of FDTD [55]. The modified k-Wave is still slow and limited in accurately modeling the acoustic attenuation in multilayer tissues. Huang et al. proposed a method to incorporate the prefactor in power law absorption model to make it as a spatially varying quantity for heterogeneous lossy media [47]. The speed problem however remains a challenge.

Deterministic ray-tracing is a method for calculating the propagation of waves through a system with regions of varying propagation velocity and absorption characteristics and explains how energy travels along a large number of lines in space between a source and a detector [56]. Kyriakou et al. employed a semi-analytical ray-tracing approach that takes the skull properties into account and allows calculation of improved effective distance-based phase corrections in transcranial ultrasound propagation [50]. Jin et al. developed a numerical model based on the ray theory for calculating the propagation of thermoacoustic waves through the skull to investigate the effects of the skull and correct for the induced phase distortion [38].

The objective of this study is to develop a fast simulation framework based on deterministic ray-tracing. The simulation takes into account the frequency-dependent attenuation and dispersion effects that occur in wave reflection, refraction, and mode conversion at the skull surface. The main advantage of the proposed framework over the current computational methods is its speed.

## 2. Materials and Method

### 2.1. Theoretical Background

In PAI, the tissue is irradiated by a short-pulsed laser beam, typically in the nanosecond range. The laser light is propagated through the tissue, absorbed by the chromophores, and converted to heat, creating an initial pressure rise via thermoelastic expansion. This initial pressure rise propagates as ultrasound waves in the tissue, referred to as PA waves [1]. The PA waves detected by ultrasonic transducers produce a weak signal. The signal is usually amplified using one or more cascaded low-noise amplifiers and digitized in a data acquisition system. The acquired signals are processed in a reconstruction algorithm to form an image [1,2].

#### 2.1.1. Mathematical Modeling of the PA Wave Propagation in a Single-Layer Heterogeneous Medium

To model the PA wave generation, let H(r,t) denote the heat energy distribution per unit volume and per unit time in the tissue generated by a laser pulse radiation. In response to the heat source, H(r,t), a thermoacoustically induced pressure wavefield, p(r,t), at the location *r* and time t≥0 in a lossless and acoustically homogeneous medium obeys the following wave equation [1,2]:

(1) $$\nabla^{2}\;p\left(r,t\right)-\frac{1}{c_{0}^{2}}\frac{\partial^{2}\;p\left(r,t\right)}{\partial t^{2}}\;=\;\frac{-\beta }{C_{p}}\frac{\partial H(r,t)}{\partial t}$$

where $c_{0}$ is thermodynamic speed of sound, β is thermal expansion coefficient, and $C_{p}$ is specific heat capacity.

*Acoustic Attenuation:* In biological tissues where acoustic properties are spatially variant, the pressure wavefield amplitude is affected by the acoustic attenuation. The attenuation is a combination of scattering and absorption in the microstructures. The tissue scattering is modeled using wave propagation theory in a random medium [57,58].

To model frequency-dependent absorption, the power law is considered [37]:

(2) $$\alpha \;=\;\alpha_{0}\omega^{y}$$

where α is the acoustic absorption, $\alpha_{0}$ is the power law prefactor, ω is the angular frequency, and *y* is the power law exponent, typically in the range of 1≤y≤1.5 for biological tissues [59].

In the case of multilayer tissue, wave reflection at the interfaces is also another important source of the amplitude attenuation that will be discussed in Section 2.1.2.

*Acoustic Dispersion:* The propagation of a sound wave through an absorbing medium is intrinsically linked with dispersion (i.e., the dependence of the phase velocity or sound speed to frequency). For power law absorption in the form of (2) with 0<y<3 and y≠1, the required dispersive phase speed or the frequency dependence of the sound speed can be shown using the Kramers-Kronig relations [60,61]:

(3) $$\frac{1}{c_{2}\left(\omega_{2}\right)}-\frac{1}{c_{1}\left(\omega_{1}\right)}\;=\;\alpha_{0}\;tan\left(\frac{\pi y}{2}\right)\left(\omega_{2}^{y-1}-\omega_{1}^{y-1}\right)$$

where $c_{1}$ and $c_{2}$ are the sound speed at two different frequencies $\omega_{1}$ and $\omega_{2}$. Here, the dispersion is defined as a variation of sound speed from a reference sound speed $c_{1}$ at a particular frequency $\omega_{1}$.

To incorporate the acoustic attenuation and dispersion into the wave Equation (1), several models have been proposed [60,61,62,63]. One of the popular models for lossy absorptive media is the one proposed by Chen et al. [63] which is based on fractional Laplacian. Treeby et al., however, agreed that Chen et al.’s model includes desired power law absorption, they showed that the model is non-dispersive; thus, they incorporated Kramers-Kronig model in their absorption model [37]. Another mathematical model to describe the wave propagation in a heterogeneous lossy medium that considers both absorption and dispersion, proposed by [37]:

(4) $$\nabla^{2}\;p\left(r,t\right)+\left(\tau \left(r\right)\frac{\partial }{\partial t}{\left(-\nabla^{2}\right)}^{\frac{y}{2}}+\eta \left(r\right){\left(-\nabla^{2}\right)}^{\frac{y+1}{2}}\right)p\left(r,t\right)-\frac{1}{c_{0}^{2}\left(r\right)}\frac{\partial^{2}\;p\left(r,t\right)}{\partial t^{2}}\;=\;\frac{-\beta }{C_{p}}\frac{\partial H(r,t)}{\partial t}$$

where τ(r) and η(r) describe the acoustic absorption and dispersion proportionality coefficients, respectively [37]:

(5) $$\tau \left(r\right)\;=\;-2\alpha_{0}\;c_{0}^{y-1}$$

(6) $$\eta \left(r\right)\;=\;2\alpha_{0}\;c_{0}^{y}tan\left(\frac{\pi y}{2}\right)$$

The reader can refer to [37] for more detailed derivation of Equation (4).

#### 2.1.2. PA Wave Propagation at Interfaces (Interaction with Skull Tissue)

**Figure 1:**
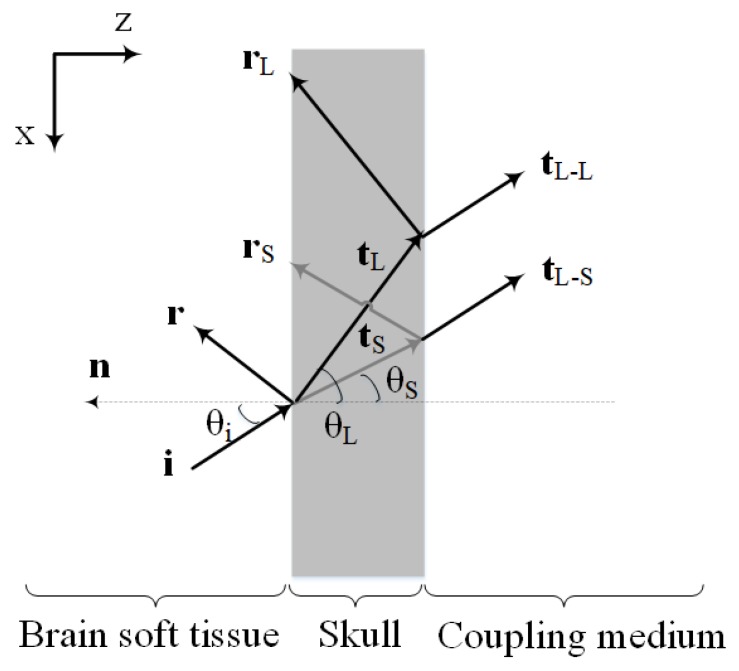
Schematic illustration of the PA wave transmission through the skull. The interfaces are assumed to be parallel with the Cartesian coordinates, where z is oriented orthogonal to the interfaces and x runs along the interface plane. Here, i denotes the incident ray direction vector, r denotes the reflected ray direction vector and t denotes the transmitted ray direction vector (the subscripts L and S refer to the longitudinal and shear rays, respectively). Also $t_{L-L}$ and $t_{L-S}$ are the longitudinal transmitted ray direction vectors generated by the incident longitudinal and shear rays in the skull, respectively. $\theta_{i}$, $\theta_{L}$ and $\theta_{S}$ are the angle of incidence, longitudinal transmittance, and shear transmittance, respectively. n is the normal vector orthogonal to the interface.

Figure 1 shows the schematic illustration of the PA wave transmission through the skull layer. As depicted, the PA waves propagating out of the brain tissue, experience reflection and refraction at interfaces between skull and tissue layers. Moreover, there will be mode conversion (i.e., longitudinal to shear wave conversion) at the inner-skull surface (i.e., the first fluid-solid interface).

Shear waves are produced when PA/ultrasound waves travel from the soft tissue to the skull [38,39,64]. The effects of shear waves in a single fluid-solid interface should become significant for angles exceeding $20^{\circ }$ with respect to the skull’s normal [38,49,65]. Yet, as demonstrated in [28] previous experimental results show that the existence of a second solid-fluid interface may reduce the effective angle at which shear waves can be excited. Therefore, the assumption that the ultrasound transcranial propagation is composed of pure longitudinal modes, is valid only for small incident wave angles, near the normal to the interface. As the incident angle begins to increase from the normal incidence, shear waves gradually dominate the transmitted acoustic waves such that for the incident angles beyond Snell’s critical angle of longitudinal wave, only pure shear waves propagate into the skull [38]. In transcranial PA brain imaging, oblique incidence is inevitable and incident acoustic waves to the skull surface may be in any arbitrary angle. Therefore, shear waves are generated in the inner-skull surface.

The transmission and reflection coefficients of each interface for an arbitrary angle of incidence can be computed using plane waves calculations. Assuming the layers be homogeneous with a constant density and speed of sound, the pressure transmission coefficients at the fluid-solid interface (considering the contribution of reflection, refraction, and mode conversion) are expressed as [64]:

(7) $$T_{L}\;=\;\left(\frac{\rho_{f}}{\rho_{s}}\right)\frac{2\;Z_{L}cos\left(2\theta_{S}\right)}{Z_{L}cos^{2}\left(2\theta_{S}\right)+Z_{S}sin^{2}\left(2\theta_{S}\right)+Z_{f}}$$

and

(8) $$T_{S}\;=\;-\left(\frac{\rho_{f}}{\rho_{s}}\right)\frac{2\;Z_{S}sin\left(2\theta_{S}\right)}{Z_{L}cos^{2}\left(2\theta_{S}\right)+Z_{S}sin^{2}\left(2\theta_{S}\right)+Z_{f}}$$

where

(9) $$Z_{L}\;=\;\frac{\rho_{s}c_{L}}{cos\theta_{L}}$$

(10) $$Z_{S}\;=\;\frac{\rho_{s}c_{S}}{cos\theta_{S}}$$

(11) $$Z_{f}\;=\;\frac{\rho_{f}c_{f}}{cos\theta_{i}}$$

here, $T_{L}$ and $T_{S}$ refer to the transmission coefficients of the longitudinal and shear waves, respectively. $\rho_{f}$ and $\rho_{s}$ are the densities of the fluid and the solid, respectively. $c_{f}$ and $c_{L}$ are the longitudinal acoustic speeds in fluid and solid, respectively and $c_{S}$ is the shear acoustic speed in the solid. $\theta_{i}$, $\theta_{L}$ and $\theta_{S}$ are the incident, longitudinal and shear refracted angles, respectively, in which the refracted angles can be obtained using Snell’s law [64].

The acoustic intensity transmission and reflection coefficients are then obtained by [64]:

(12) $$I_{L}\;=\;\frac{\rho_{f}tan\theta_{i}}{\rho_{s}tan\theta_{L}}|T_{L}{|}^{2}$$

(13) $$I_{S}\;=\;\frac{\rho_{f}tan\theta_{i}}{\rho_{s}tan\theta_{S}}|T_{S}{|}^{2}$$

(14) $$I_{r}\;=\;1-\left(I_{L}+I_{S}\right)$$

where $I_{L}$ and $I_{S}$ are the intensity transmission coefficients of the longitudinal and shear waves, respectively and $I_{r}$ is the intensity-reflection coefficient.

Equations (12)–(14) relate the acoustic intensity transmission and reflection coefficients with the properties of the media and the angle of incidence. It is noteworthy that the expressions for transmittance in Equations (7) and (8), are obtained by taking into account the boundary condition of the continuity of normal and shear stress components and normal velocities at the interface. The frequency dependence of these acoustic transmission coefficients is also due to the frequency dependence of the longitudinal and shear speeds of sound in the media, which is described by dispersion relation in Equations (3).

The values obtained by this analysis are then used as the input for the next interface, for which a similar approach is taken into account to determine the transmission coefficients for the solid-fluid interface. As depicted in Figure 1, the incident shear waves are converted to the longitudinal waves in transmitting from the skull to the coupling medium. Therefore, only the longitudinal waves eventually propagate to the coupling medium and we only have the longitudinal transmission coefficients at the second solid-fluid interface.

### 2.2. Simulation Framework

Obtaining an exact solution from (4) is complicated when dealing with media such as the skull bone and brain soft tissue; Particularly, due to the high acoustic impedance mismatch, irregular shape, and non-uniform thickness of the propagation medium. The most commonly used numerical methods for solving acoustic equation are the methods based on finite-difference, finite-element, and boundary-element methods [36,44,50,51,52,53,54]. However, in the case of modeling high-frequency waves, they are slow especially for large 3-D media. k-space pseudo-spectral time-domain solution is the modified version of the standard FDTD model in which a periodic function known as the k-space operator is introduced [55]. Even with these modifications, k-space methods are computationally expensive especially when the grid size is fine.

**Figure 2:**
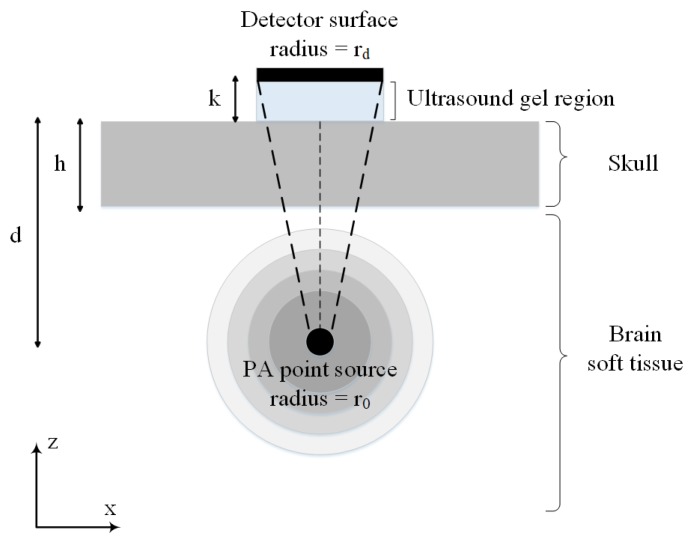
2-D illustration of a simple model of skull used in our simulations. The model consists of a skull bone with a thickness of *h* located above the brain tissue. The spherical PA imaging target with the radius of $r_{0}$ is located at a depth of *d* below the skull layer and a flat ultrasound transducer with an element diameter of $2r_{d}$ is remained in contact with the outer-skull surface and the coupling gel.

Figure 2 shows the simplified 2-D illustration of the synthetic skull that is used in our simulations. In this model, broadband spherical acoustic waves are generated by a PA point source. The waves are transmitted through the skull and detected by a transducer. A layer of skull bone with a thickness of *h* is considered above a brain tissue layer with a thickness of *T*. The PA point source is modeled as a sphere with the radius of $r_{0}$ at a depth of *d* below the skull layer. The skull was acoustically coupled to a transducer using ultrasound gel with a thickness of *k*. A flat ultrasound transducer with an element diameter of $2r_{d}$ is remained in contact with the outer-skull surface and the coupling gel. Also, the solid acceptance angle of the detector is indicated by the dashed lines which becomes larger as the point source becomes closer to the inner-skull surface, hence the conversion of longitudinal waves to shear waves becomes significant. We are interested in both longitudinal and shear waves and used the transmission functions given in (7) and (8) for the simulation of the transmitted pressure at the skull–tissue interface. All acoustic properties used in the simulations are listed in Table 1.

The imaging target is a spherical absorber inside the brain tissue (as shown in Figure 2). It is assumed that the tissue is illuminated with a Gaussian laser pulses to generate the initial pressure rise at the location of the absorber. The absorbing compartments acted as sources of acoustic waves that isotropically propagate waves. The spatial and temporal distribution of such acoustic pressure is found via convolution of the PA impulse response with the Gaussian laser function, written as [67]:

(15) $$p\left(r,t\right)\;=\;\frac{-\beta E_{a}}{2\pi^{3/2}C_{p}\tau_{e}^{2}\;r}\left(\frac{t-\tau }{\tau_{e}}\right)e^{\frac{-1}{2}{\left(\frac{t-\tau }{\tau_{e}}\right)}^{2}}$$

(16) $$\tau =\frac{r}{c_{0}}$$

(17) $$\tau_{e}\;=\;\sqrt{\tau_{a}^{2}+\tau_{l}^{2}}$$

(18) $$\tau_{a}=\frac{r_{0}}{c_{0}}$$

where $E_{a}$ is the absorbed optical energy, $r_{0}$ is the radius of the spherical absorber, $\tau_{a}$ is half of the acoustic transit time across the spherical absorber and $\tau_{l}$ is the laser pulse duration. All the other parameters are defined in the Section 2.1.1.

The simulator is based on parallel executable ray-tracing core written entirely in Java. For the means of advanced signal processing purposes, the simulator is interfaced with MATLAB R2016a which can be used arbitrarily. The core of the proposed PA wave propagation simulator is consisted of the following subsections.

#### 2.2.1. Ultrasound Ray Vector Space

To simulate the forward propagation of the generated PA waves through the brain tissue and skull, initially and as the only real discretization, the simulator builds a mesh of arbitrary size (depending on the transducer dimension) around the transducer and discretizes this mesh into small cells (much smaller than the acoustic wavelength, i.e., <<1 mm). Since, the main focus of the current study was to develop a fast skull modeling algorithm based on deterministic ray-tracing, we considered some simplifications in modeling such as assumption of the smooth surface of the skull in a very small area comparable to the acoustic wavelength [68]. Considering non-smooth skull surface, i.e., integrating the results of this study in a finite-element model, will be the next step of this study which will be addressed as future work. In this study, a mesh of size 100 × 100 was built around the transducer surface. Then, the algorithm calculates the skull entry/exit (i.e., intersection) points of the rays between the desired target and each element’s surface center of the discretized region around the transducer. It then uses that information to draw the imaginary vectors which will guide the actual rays of ultrasound wave to the transducer surface. According to the discretization carried out in this paper, 10${}^{4}$ rays will be launched from the target to the transducer surface at each time and frequency. We treat these rays of ultrasound wave like mutually independent vectors in time-frequency-space dimension which unless necessary do not interact to each other.

The simulation of the wave propagation was achieved by time-frequency decomposition of the incident wave using the short-time Fourier transform (STFT) algorithm [69], followed by the determination of the ray paths (i.e., ray direction vector), and then tracing every single sample ray of each frequency and subjecting them to the tissue absorption and dispersion along with the acoustic reflection, refraction and mode conversion occurred at boundaries based on the formulation presented in our model. Since, in ray-tracing simulation model we need to take into account every single-frequency ray at every single time it might appear, the joint time-frequency information of the signal containing multiple time-varying frequencies are required. Therefore, we performed STFT rather than standard Fourier transform, in which it simultaneously provides temporal information and spectral information. In this study, 64 frequencies are modeled. At the inner-skull boundary, the incident wave is split into a reflected wave, a transmitted longitudinal wave, and a transmitted shear wave (Figure 1). The amplitudes of the transmitted waves can be determined relative to the incident wave using method outlined by White [64] and presented in Equations (7) and (8). Also, the reflected and refracted direction vectors in this fluid-solid interface can be calculated via [70]:

(19) $$r=i+2cos\theta_{i}n$$

(20) $$t=\frac{n_{1}}{n_{2}}i+\left(\frac{n_{1}}{n_{2}}cos\theta_{i}-\sqrt{1-sin^{2}\theta_{t}}\right)n$$

with:

(21) $$cos\theta_{i}\;=\;-i.n$$

where r and t are the direction vectors of the reflected and longitudinal or shear transmitted rays, respectively. i is the incident ray direction vector, n is the normal vector orthogonal to the interface and pointing towards the first medium, $n_{1}$ and $n_{2}$ are the refractive indices of the two media, and $\theta_{i}$ and $\theta_{t}$ are the angle of incidence and longitudinal or shear transmittance, respectively.

The simulator is capable of arbitrary number of reflections for each ray. In this simulation we have set the maximum allowed reflection number to 20. Rays are supposed to die if they go out of simulation area or if they are received at the transducer surface; of course, rays reflected from the transducer are not dead.

#### 2.2.2. Modeling of Impulse Response

The developed simulator does not simulate every single ray every single time it might appear. Instead, for each frequency given, it makes a pattern of results as an impulse response, if a sample ray of unit amplitude is launched at time t = 0 (i.e., Dirac delta function) at every direction vector given. This impulse sample ray propagated at every direction vector in each frequency, results in an array of detected rays at the transducer surface in different times and amplitudes. These differences in amplitude values and timings, are caused by different frequency-dependent behaviors of attenuation, dispersion, reflection, and transmission at the inner-skull surface. Please note that the amplitudes received at the same times should be added for each frequency. It is worth noting that in accordance to frequency response characteristics of the transducer, different responsitivity for different frequencies can be applied for the detected rays at the transducer surface. This is achieved only because in the ray vector space, we maintain vector of each ray till its death or arrival in the transducer hence enabling the simulator to apply arbitrarily realistic characteristics of the transducer to the simulation setup.

#### 2.2.3. Convolution

The simulator is fed with the STFT matrix of the incident wave at the target surface, containing time, frequency, and amplitude tuple for real and imaginary values. The key to fast implementation of our simulator is that after the above-mentioned impulse response patterns are made, we only need for each frequency the input signal of the simulator is convolved with the corresponding impulse response to get the STFT matrix at the transducer location. Finally, the detected STFT matrix is inverse transformed to give the pressure over the measurement plane.

## 3. Validation

### 3.1. Numerical Validation

The simulation results obtained from our simulator is compared to those obtained from k-Wave. K-Wave is a MATLAB toolbox which uses a k-space corrected pseudo-spectral time-domain algorithm to solve the coupled first-order conservation equations for the simulation of propagating acoustic waves in heterogeneous media. Details of implementation of the k-space pseudo-spectral algorithm using the k-Wave MATLAB toolbox can be found in [55].

The model structure shown in Figure 2, is initially simulated with the k-space algorithm to detect the PA signal by the ultrasound point transducer which is placed just above the absorber. In this regard, the k-space lossy elastic model has been used to simulate the compressional and shear waves propagation in viscoelastic medium in which absorption and dispersion are present. Subsequently, simulations of the same structure are performed using our proposed framework. Six distinct structures are simulated to assess the agreement between the two simulators with the study of the effect of skull thickness and target location relative to the skull layer. Three of the simulations are performed with constant target depth, i.e., *d* = 1.7 cm, and different skull thickness of 1 mm, 4 mm, and 7 mm. In the other three simulations the imaging target is moved away from the inner-skull surface 1 cm to 3 cm, while the skull thickness remains constant at *h* = 7 mm. All acoustic properties of the simulation medium including speed of sound, density, and attenuation are the same as the ones in Table 1.

To further quantify the agreement between the two simulated PA signals, the normalized standard deviation (NSD) is calculated as:

(22) $$NSD\left(\%\right)=\frac{l^{2}\left(|PA_{ray-tracing}|-|PA_{k-Wave}|\right)}{l^{2}\left(|PA_{k-Wave}|\right)}\times 100$$

with $l^{2}$ norm being the square root of the average of the squared differences from the mean values for the respective signal samples.

### 3.2. Analytical Validation

We also performed analytical validation of the results based on the calculation of the longitudinal and shear intensity transmission coefficients, $I_{L}$ and $I_{S}$. Incident acoustic plane waves are assumed on the inner-skull surface at different incidence angles $\theta_{i}$. The longitudinal and shear intensity transmission coefficients are calculated both analytically, through Equations (12) and (13) at the reference frequency and, numerically, through the amplitudes of the incident and transmitted signals simulated by our proposed simulator. Three such simulations are performed where the incidence of a plane wave from a soft tissue on the inner-skull surface is modeled at different incidence angles of 0${}^{\circ }$, 15${}^{\circ }$, and 30${}^{\circ }$. All acoustic properties used in analytical solution and numerical simulation including speed of sound and density are the same as the ones in Table 1.

To further quantify the agreement between the two analytically and the numerically determined coefficients, two error measures are introduced as:

(23) $$E_{L}\left(\%\right)=\frac{I_{L(ray-tracing)}-I_{L(analytical)}}{I_{L(analytical)}}\times 100$$

(24) $$E_{S}\left(\%\right)=\frac{I_{S(ray-tracing)}-I_{S(analytical)}}{I_{S(analytical)}}\times 100$$

where $E_{L}$ and $E_{S}$ denote the estimated errors in the numerically obtained longitudinal and shear intensity transmission coefficients, respectively, relative to the reference values, i.e., analytically determined intensity transmission coefficients.

### 3.3. Experimental Validation

#### 3.3.1. Experimental Setup

**Figure 3:**
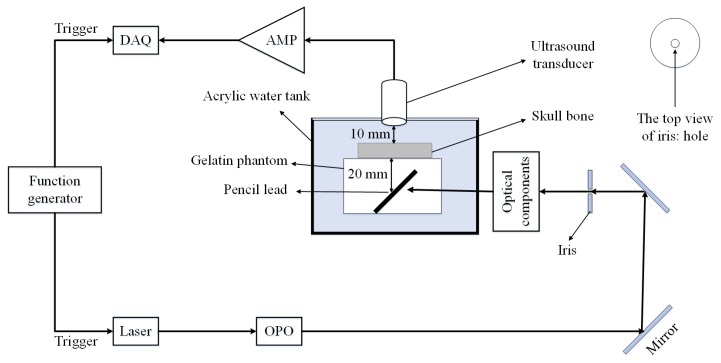
Schematic of the experimental setup. DAQ: Data acquisition system, AMP: Amplifier, OPO: Optical parametric oscillator.

The proposed model is validated using the experimental setup depicted in Figure 3. It consists of a flat ultrasound transducer (Technisonic ISL-0504-GP research immersion transducer, 5 MHz, element diameter: 0.5 inch, and 6 dB fractional bandwidth: 36%), a data acquisition system including a processing unit (National Instrument FPGA-based system, 16 Channels, 14 Bit, max sampling rate: 250 MS/s), a low-noise amplifier (ZFL500LN, Minicircuit, New York, NY, USA) with the gain of 24 dB, and a DC power supply of 15 V, and 60 mA. The light illumination is performed using 7 ns duration pulses and 30 Hz repetition rate generated by an Nd:YAG laser (Spectraphysics Q-Switched laser) [71,72]. Other parts of the system are illustrated in Figure 3.

#### 3.3.2. Phantom Preparation

The phantom is made using 7% gelatin in distilled water. A piece of pencil lead with diameter of 0.2 mm and $45^{\circ }$ orientation is placed inside the phantom before the gelatin solidified. This will accurately mimic the PA point source. The imaging target depth from the inner-skull surface is 20 mm and the distance between the outer-skull surface and the transducer (coupling medium surrounding the skull) is 10 mm.

**Figure 4:**
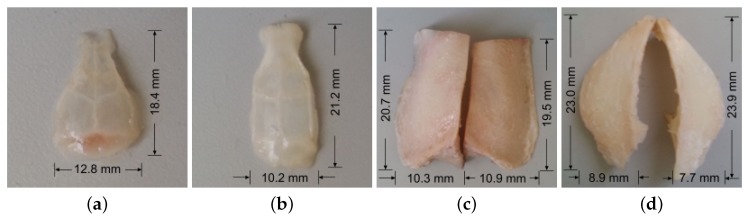
Photographs of (**a**) mouse skull bone, (**b**) rat skull bone, (**c**) dog frontal skull bone, (**d**) dog parietal skull bone.

We then test the effects of mouse, rat and mesocephalic dog skull bones (see Figure 4) on PA signal for further evaluation of our model. The thickness of the mouse skull, rat skull, dog frontal and parietal skulls are 0.5 mm, 1 mm, 4.5 mm, and 9 mm, respectively. The skull thicknesses are measured using Vernier scale. All the bones are cleaned and washed to remove excess tissue and blood. The skulls are stored in phosphate buffered saline (PBS) and refrigerated during storage. Wayne State University Animal Care and Use Committee approved the study.

To examine the effects of the skull on the received PA signal, two data sets are acquired: the first one with the skull bone placed in the acoustic propagation pathway between the phantom and the transducer and the second one without it. The laser excitation wavelength in this experiment is set to 650 nm. The laser had a circular beam shape with a diameter of 10 mm. We focused laser light to a pencil lead [73]. The laser reflects off a mirror (“BB1-E02-⌀1” Broadband Dielectric Mirror, Thorlabs, Newton, NJ, USA) and then passes through an Iris (SM1D12C - SM1 Graduated Ring-Actuated Iris Diaphragm, Thorlabs, USA). Then the laser light passes a convex lens (AC254-100-A1, Thorlabs) with a 100-mm focal length and 50-mm diameter to focus the laser beam on the objective lens. An objective lens (40×, Newport Corporation, Irvine, CA, USA) with the NA of 0.65 and a magnification of 40 is used to spherically shape the beam. The beam is then focused onto the pencil lead through another convex lens (AC254-060-A1, Thorlabs) with 60-mm focal length and 50-mm diameter. We implemented a transmission mode PAT setup. The transducer is placed on top of the sample perpendicular to the surface of the skull tissue.

## 4. Results

**Figure 5:**
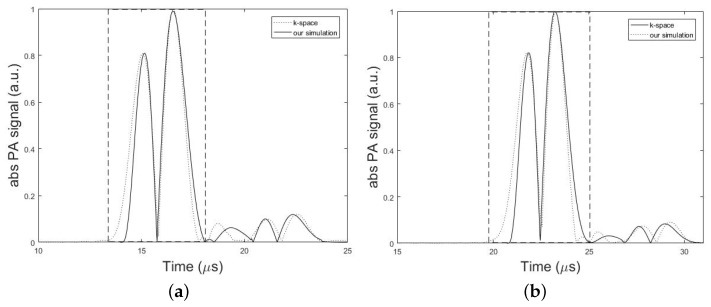
Numerical validation of the proposed method versus the k-space algorithm. Absolute PA signal profiles simulated by k-space algorithm (dotted line) and our simulation method (solid line). Dashed rectangles show the main bipolar pulse of the simulated PA signal. In these simulations, the setup in Figure 2 with the parameters, *h* = 7 mm, *k* = 10 mm, $r_{0}$ = 1 mm, $r_{d}$ = 0.025 inch, and *T* = 5 cm is used. (**a**) Minimum error of 0.11% obtained for *d* = 1.7 cm. (**b**) Maximum error of 0.25% obtained for *d* = 2.7 cm.

The experimental results as well as the results obtained from our simulations are presented in this section. Figure 5, compares the absolute amplitudes of two simulated PA signals from a single spherical absorber using k-space algorithm and our simulator in two different conditions corresponding to the minimum and maximum error obtained from the simulations. Dashed rectangles in Figure 5 show the main bipolar pulse of the PA signal. In Figure 5a, the setup in Figure 2 was used with the parameters, *h* = 7 mm, *d* = 1.7 cm, *k* = 10 mm, $r_{0}$ = 1 mm, $r_{d}$ = 0.025 inch, and *T* = 5 cm and the minimum error of 0.11% was obtained from the analysis of NSD value. In Figure 5b, simulations of the same structure were also performed only by changing the value of parameter *d* to 2.7 cm. In this case, the maximum error of 0.25% was obtained from the analysis of NSD value. In overall, a good agreement between two models was obtained with an average error of 0.17%. The results can be seen in Table 2 and Table 3.

To further verify the proposed simulator, the analytical validation is also performed based on the calculation of the intensity transmission coefficients. The results of longitudinal and shear intensity transmission coefficients obtained numerically and analytically are given in Table 4. These results are in a good agreement with errors in the order of 0.00% to 4.51%, thus verifying analytically our proposed algorithm. Please note that in numerical simulation by the ray-tracing model for each incidence angles of 0${}^{\circ }$, 15${}^{\circ }$, and 30${}^{\circ }$, only one ray was launched to the inner-skull surface at each frequency and then the transmitted intensity of the longitudinal and shear waves were individually calculated at this interface. By this way, we have two individual STFT matrix corresponding to the transmitted longitudinal and shear waves at the inner-skull surface. These two STFT matrix are now inverse transformed to give the longitudinal and shear waves individually just above the inner-skull surface. Finally, the longitudinal and shear intensity transmission coefficients are calculated by dividing the intensity of the corresponding transmitted waves to the intensity of the incident waves at the inner-skull surface.

**Figure 6:**
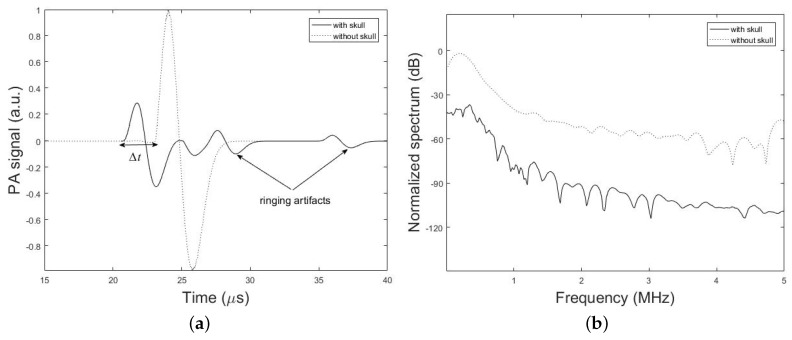
Simulation results. (**a**) PA signals simulated by our simulation method in the presence and absence of a skull tissue. The setup in Figure 2 with the parameters, *h* = 7 mm, *d* = 2.7 cm, *k* = 10 mm, $r_{0}$ = 1 mm, $r_{d}$ = 0.25 inch, and *T* = 5 cm is used. (**b**) Corresponding frequency spectrum of the simulated PA signals.

Figure 6, demonstrates the time-domain PA signals and the corresponding normalized frequency spectrum simulated by our method with the presence and absence of the *h* = 7 mm thick skull above the brain tissue layer. The spherical PA source with the radius of $r_{0}$ = 1 mm is located at the depth of *d* = 2.7 cm from the ultrasound coupling gel with thickness of *k* =10 mm. The ultrasound transducer with the element diameter of $2r_{d}$ = 0.5 inch is used in contact with the coupling gel. It can be seen that the peak of the transcranially recorded PA signal is significantly lower in amplitude compared to that of without skull. It is also shown that the presence of the skull in the acoustic signal propagation path, makes the signal broader. The effects of time shift and multiple ringing artifacts on the PA signal due to the presence of skull are shown in Figure 6a.

Amplitude attenuation and broadening of the signal are attributed to the frequency-dependent acoustic attenuation and dispersion of the skull which acts as a low-pass filter according to (2) and (3). The explanation for such distortions is as follows: due to the dispersion of the skull, different frequency components of the PA wave would travel in different speeds and hence they will arrive at the transducer at different times (or phases) which in turn would result in a broader signal compared to the undisturbed signal in the absence of the skull. Furthermore, because of the frequency-dependent attenuation of the skull higher frequencies are significantly attenuated and have a lower transmission efficiency; hence the frequency spectrum of the transcranially recorded signal becomes narrower in bandwidth compared to the undisturbed signal, i.e., broadening of the time-domain signal. Figure 6b shows that 3 dB bandwidth of the signal is 0.23 MHz and 0.11 MHz in the absence and presence of the skull, respectively, which confirms the narrower bandwidth of the signal in the presence of the skull and therefore the low-pass filtering characteristic of the skull. Also, the significantly higher speed of sound in the bone as compared to brain soft tissue makes PA waves travel faster through the skull and detected earlier at the detector location. As a result, this leads to time shift in the transcranially recorded signal relative to the undisturbed signal marked by Δt in Figure 6a. The last signal distortion occurs due to the reflection of PA waves from skull–tissue interfaces with the resultant time-domain ringing artifacts at the end of transcranially recorded signal.

**Figure 7:**
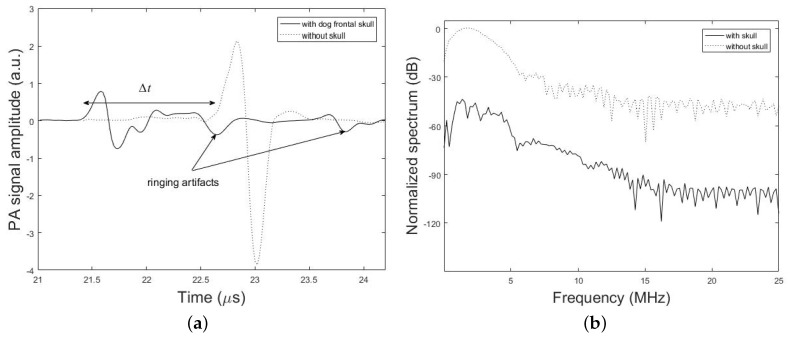
Experimental results. (**a**) PA signal amplitude obtained with and without dog frontal skull bone. The skull bone was placed in the detection path. (**b**) Corresponding frequency spectrum of the experimental PA signals.

Figure 7 shows PA signal profiles obtained from the imaging target with and without the dog frontal skull sample. The effect of time shift (6.31%), attenuation (74.58%), dispersion (13.33% signal broadening) and ringing artifact on the PA signal is shown in this figure. The percent values for each parameter in these results is calculated via the difference between the desired parameter at two conditions with and without the skull, that is divided into the parameter without the skull. It is noteworthy that the differences in the experimental and simulation signal shapes and time scales arise from the differences in the setup used in experiments and simulations.

**Figure 8:**
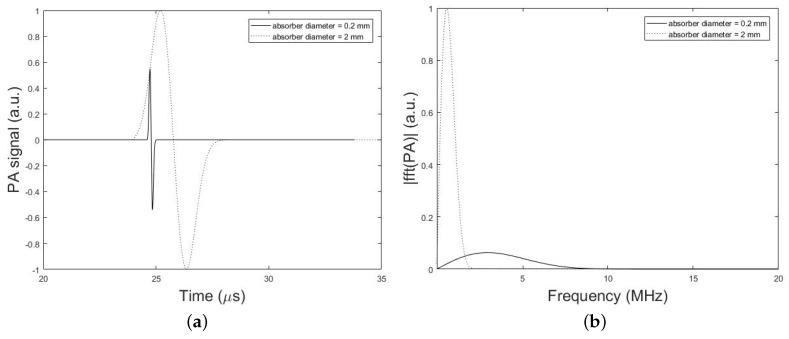
Simulation results. (**a**) PA signals simulated by our simulation method in the absence of the skull tissue for two different diameters of spherical absorber 2 mm (dotted line) and 0.2 mm (solid line). The setup in Figure 2 with the parameters, *h* = 0 mm (i.e., without skull), *d* = 2.7 cm, *k* = 10 mm, $r_{0}$ = 1 mm or 0.1 mm, $r_{d}$ = 0.025 inch, and *T* = 5 cm is used. (**b**) Corresponding normalized magnitude of the frequency spectrum of the simulated PA signals.

The frequency bandwidth of the emitted PA wave is also affected by the PA absorber dimension. Figure 8, compares the time-domain PA signals and the corresponding normalized magnitude of the frequency spectrum from a single spherical absorber with two different diameters of 2 mm and 0.2 mm. In these simulations, the setup in Figure 2 is used and the parameters set to, *h* = 0 mm (i.e., without skull), *d* = 2.7 cm, *k* = 10 mm, $r_{0}$ = 1 mm or 0.1 mm, $r_{d}$ = 0.025 inch, and *T* = 5 cm. It can be seen that the PA signal originating from the smaller absorber is narrower in time-domain while emits broader waves in the frequency domain compared to the larger absorber. As depicted in Figure 8, the signal intensity attenuation largely depends on the size of the absorber that generates the original signal, so that the frequency content generated by the small-sized absorber is attenuated to a much greater extent as compared to signal from large absorber. Thus, it is expected that the stronger amplification will be needed for signals originating from small absorbers in correction algorithm.

To understand the effect of skull thickness on the PA signal distortions, we explored the amplitude attenuation, signal broadening, and signal time shift as a function of the skull thickness. In these simulations, the setup in Figure 2 is used with the parameters, *d* = 1.7 cm, *k* = 10 mm, $r_{0}$ = 1 mm, $r_{d}$ = 0.25 inch, and *T* = 5 cm. The thicknesses of the skull are as follow; 0.5 mm, representing mouse skull thickness, 1 mm, representing rat skull thickness, 1.5 mm, representing neonatal skull thickness [74], 4.5 mm, representing dog frontal skull thickness, 5.98 mm, and 7.68 mm, representing human frontal skull thickness [75], 9 mm, representing dog parietal skull thickness, and 9.61 mm, representing human frontal skull thickness [75].

**Figure 9:**
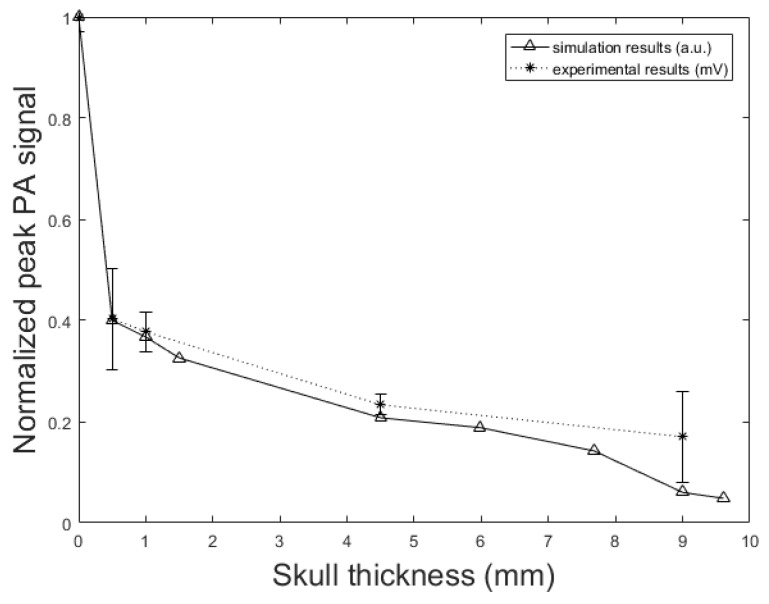
PA signal amplitude attenuation versus skull thickness. Simulation results (solid line) and experimental phantom results (dotted line). In simulation, the setup in Figure 2 with the parameters, *d* = 1.7 cm, *k* = 10 mm, $r_{0}$ = 1 mm, $r_{d}$ = 0.25 inch, and *T* = 5 cm is used. The thicknesses of the skull are as follow; 0.5 mm, representing mouse skull thickness, 1 mm, representing rat skull thickness, 1.5 mm, representing neonatal skull thickness, 4.5 mm, representing dog frontal skull thickness, 5.98 mm, and 7.68 mm, representing human frontal skull thickness, 9 mm, representing dog parietal skull thickness, and 9.61 mm, representing human frontal skull thickness.

Figure 9 shows the normalized PA signal amplitude versus different skull thicknesses. The decline in the normalized PA peak signal with the increase of the skull thickness is also seen in the experimental results. In the experimental setup, when the skull is placed in the propagation pathway, the normalized PA signal amplitude decreased from 0.41 mV to 0.17 mV with increasing skull thickness from 0.5 mm to 9 mm. This means that the amplitude attenuation increased from 59% to 83%; the rate of increase is 60% to 95.17% in our simulation with increasing skull thickness from 0.5 mm to 9.61 mm. The error bars in experimental data were calculated by repeating the experiments 10 times.

**Figure 10:**
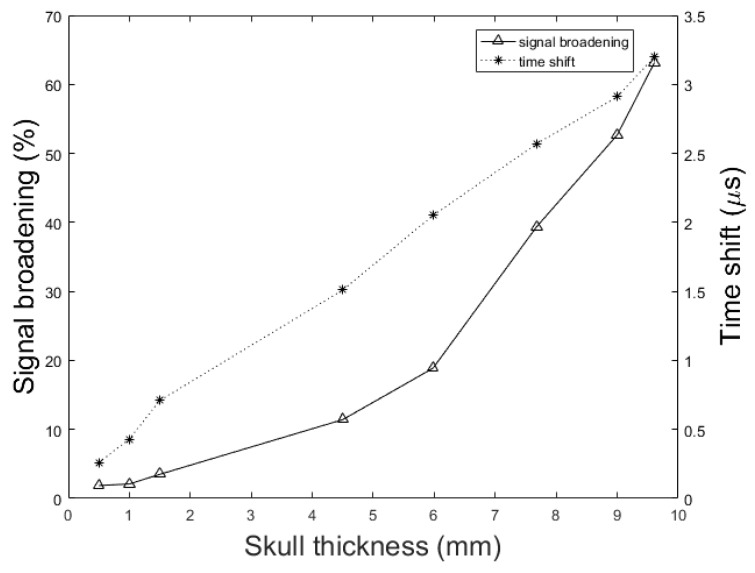
Simulation results of the effect of skull thickness on the signal broadening (solid line) and time shift (dotted line) of transcranially recorded signal relative to the undisturbed signal. In these simulations, the setup in Figure 2 with the parameters, *d* = 1.7 cm, *k* = 10 mm, $r_{0}$ = 1 mm, $r_{d}$ = 0.25 inch, and *T* = 5 cm is used. The thicknesses of the skull are as follow; 0.5 mm, representing mouse skull thickness, 1 mm, representing rat skull thickness, 1.5 mm, representing neonatal skull thickness, 4.5 mm, representing dog frontal skull thickness, 5.98 mm, and 7.68 mm, representing human frontal skull thickness, 9 mm, representing dog parietal skull thickness, and 9.61 mm, representing human frontal skull thickness.

Figure 10 shows the signal broadening for different thicknesses of the skull. It can be seen that the signal broadening increased from 1.86% to 63.17% with increasing skull thickness from 0.5 mm to 9.61 mm. Here, the signal broadening is defined as the difference between the duration of the PA signal under two circumstances; with and without the skull tissue. The duration of a signal is calculated as the difference between the signal incidence time and the signal suppression time. Also, in Figure 10 we see that the resulting time shift due to the difference between the speed of sound, increased from 0.26 μs to 3.2 μs with the same change in skull thickness. The time shift in the transcranially recorded signal relative to the undisturbed signal in model structure used in this study (Figure 2) can be calculated by:

(25) $$\Delta t\;=\;\frac{h}{c_{0}}-\frac{h}{c_{skull}}$$

where *h* is the skull thickness, $c_{0}$ is the speed of sound in the brain soft tissue, and $c_{skull}$ is the longitudinal speed of sound in the skull. Using the thickness of 4.5 mm skull bone and with the reported values of sound speed in soft tissue and skull in literature, i.e., 1500 (m/s) and 2900 (m/s) respectively, the time shift is calculated as 1.44 μs, which is confirmed by our numerical simulation result of 1.51 μs.

**Figure 11:**
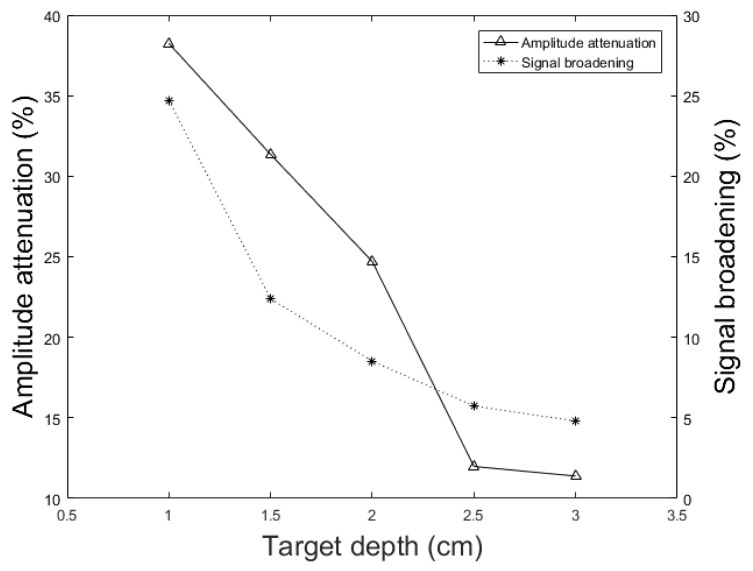
Simulation results of the effect of different target locations on the PA peak signal amplitude attenuation (solid line) and signal broadening (dotted line). In these simulations, the setup in Figure 2 with the parameters, *h* = 7 mm, *k* = 10 mm, $r_{0}$ = 1 mm, $r_{d}$ = 0.25 inch, and *T* = 5 cm is used. The imaging target is moved away from the inner-skull surface from 1 cm to 3 cm.

In addition to the skull thickness, we explored the effect of the distance between the imaging target and the inner-skull surface on the skull-induced acoustic attenuation and signal broadening. Figure 11 shows that the amplitude attenuation decreased from 38.24% to 11.39% with increasing the distance between target and inner-skull surface from 1 cm to 3 cm for constant thickness of skull, i.e., 7 mm. In Figure 11 we also see that the signal broadening effect decreased from 24.71% to 4.80% with the same change in target-skull distance. That means that the farther away the imaging target is from the inner-skull surface the less acoustic attenuation and phase distortion caused by the skull layer. In cases, where the imaging target is very close to the inner-skull surface, the skull-induced distortions become significant and the reconstructed image will significantly be distorted. As can be seen in Figure 9, Figure 10 and Figure 11, under the assumption that the layers between the imaging target and the transducer are homogeneous layers with a constant density and speed of sound, the skull thickness and target location relative to the inner-skull surface are the main parameters affecting the amplitude and phase of the received PA signal. Other parameters such as the detector’s geometry, its central frequency and its relative position to the skull can affect the skull-induced distortions which will be addressed as a future work for this research.

The running time for our simulation platform is far smaller than that of k-Wave especially when the grid size is fine. In [55], it has been demonstrated that k-space methods have inherent computational advantages over analogous pseudo-spectral or finite-difference methods. The computation speed of k-Wave calculated for different grid size using different data types and parallel processing, demonstrated the out-performance of the k-Wave algorithm. Therefore, we compared our simulator only to k-Wave and did not compare it to other computational methods.

The computation speed of the proposed framework is linearly proportional to the maximum frequency supported by the sampling rate of the signal. It also depends linearly to the number of frequencies which are modeled. In contrast, the computational speed of the k-Wave is heavily dependent on the accuracy of the grid spacing and therefore the maximum frequency supported by the computational grid at wave propagation space. Table 5 compares the running time of k-Wave with our simulation method for different maximum frequency supported by the grid space to model the desired spatial domain size of 64${}^{3}$ mm${}^{3}$. In these simulation, the setup in Figure 2 is used with the parameters, *h* = 0.7 cm, *d* = 1.7 cm, *k* = 10 mm, $r_{0}$ = 1 mm, $r_{d}$ = 0.025 inch. In k-Wave the grid spacing must be sufficiently small to ensure that the highest frequency of interest can be supported. For example, to support maximum frequency of 1 MHz, the grid size of 0.75 mm is chosen and therefore the computational domain size is 86${}^{3}$ grid points for spatial domain size of 64${}^{3}$ mm${}^{3}$. It takes about 849.45 s to simulate this structure by k-Wave on a common dual-core Intel Core i5 system with 8 GB of RAM in double precision. Decreasing the grid size to 0.5 mm (i.e., the computational size of 128${}^{3}$ grid points) for the simulation of maximum frequency supported of 1.5 MHz, results in increasing the running time of k-Wave by approximately 3 times and it also increased more than 20 times when the $f_{max}$ increased just to 3 MHz. No data are available for the k-Wave computation using maximum frequency supported of 5 MHz and 7.5 MHz, as these exceeded the available memory of the particular card used for the comparison. As can be seen, the running time of our method is far smaller than that in k-Wave and changes linearly with the maximum frequency supported and number of frequencies modeled.

In another investigation in Table 6, the minimum running time of k-Wave is compared with our method for 64 frequencies and sampling rate of 50 MHz for the simulation of different skull thicknesses with different decimal precision at two target depths of 1.7 cm and 3.7 cm in model structure used. For example, for the simulation of *h* = 7 mm with computational domain size of 64${}^{3}$ grid points for target depth equal to 3.7 cm, the grid size of 1 mm is chosen for maximum speed of computation and k-Wave running time is about 168.29 s. Decreasing the grid size to 0.5 mm for the simulation of *h* = 7.5 mm at same target depth, results in increasing the running time of k-Wave by approximately 15 times and increased more than 100 times when the grid size decreased to 0.25 mm. As discussed, the running time of our method remains almost constant for the fixed sampling rate and number of frequencies modeled. Although for large grid sizes the k-Wave simulation is faster than or comparable to our simulation method, increasing the grid size results in a reduction in the maximum frequency supported by the grid space. For example, maximum frequency supported by grid size of 1 mm is less than 1 MHz which is not accurate enough for simulating a higher-frequency component generated by PA signal. Therefore, the proposed simulator significantly outperformed the k-Wave method when large 3-D volume and fine grid size is considered in the simulation to support higher-frequency components of the PA signal. For example, as can be seen in Table 6, for the simulation of *h* = 7.5 mm and *d* = 3.7 cm, with the grid size of 0.5 mm, results 128${}^{3}$ elements and takes about 2533.10 s by k-Wave, while the running time by our proposed method is only 135.21 s; i.e., 18 times faster. Decreasing the grid size to 0.25 mm (i.e., the computational size of 256${}^{3}$) for the simulation of *h* = 7.25 mm, increases the factor to more than 100 times. The fast implementation of our simulator is as a result of modeling the impulse response for each frequency and then implementing the convolution of each time-frequency component of the input signal with the corresponding frequency impulse response to get the STFT matrix of the output signal at the transducer location.

## 5. Discussion

The main purpose of this work was to develop a fast and accurate numerical simulation framework to explore various acoustic distortions introduced by skull on a PA wave. The proposed simulation framework which is based on the deterministic ray-tracing principles, has computational advantages over analogous k-Wave or FDTD methods. This is due to the impulse response modeling for each frequency and then implementing the convolution of each time-frequency component of the input signal with the corresponding frequency impulse response, instead of simulating every single-frequency ray at every single time it might appear.

For the sake of comparison in terms of computational complexity, we assumed that the medium was a set of acoustically homogeneous layers. The accuracy of the simulator was also validated by k-Wave and analytical method. The simulator was then used to study the major skull-distorting effects on PA waves. The effect of skull thickness and the distance between the imaging target to the inner-skull surface were comprehensively investigated. The acoustic low-pass filtering effects of the skull were assessed showing the significant loss of high-frequency components (see Figure 6b).

We showed that the skull thickness and the location of the target relative to the inner-skull surface were the two major causes of attenuation and distortion; targets closer to the inner-skull surface and thicker skulls distort the PA signal more significantly (see Figure 9, Figure 10 and Figure 11).

Time shift in the PA signal relative to that in the undisturbed signal is attributed to the difference in speed of sound in the bone as compared to that in soft tissue, which can be used as an indicator of the skull thickness in the ultrasound propagation pathway (see Figure 6a).

Another observation was the signal distortion occurred at the skull–tissue interface due to the reflection of PA waves, along with the resultant time-domain ringing artifacts at the end of the signal (see Figure 6a). Effect of these multiple reflections of the PA wave at the inner and outer surfaces of the skull may mistakenly be interpreted as signals generated by optical absorbers located deeper inside the brain, which further deteriorate the reconstructed images.

The existence of a second solid-fluid interface may reduce the effective angle at which shear waves can be excited. Therefore, mode conversion plays a major role in distorting the PA wave, with increasing the incident angle from the normal incidence. Mode conversion causes asymmetric attenuation of the PA signal and hence, bipolar asymmetry in the shape of the PA signal (see Figure 6a). If the shear waves are ignored in the compensation process, many Fourier components in the PA signal will be mis-estimated. Also, due to the larger attenuation of shear waves compared to that of longitudinal waves, the additional contributions of these waves in multiple reflections within the skull bone are negligible. As a result, one can say that the multiple reflection artifacts arise mainly because of longitudinal waves.

We demonstrated that the frequency bandwidth of the emitted PA wave and consequent frequency-dependent attenuation were also affected by the PA absorber size such that the smaller absorbers emitting broader band waves (see Figure 8). Also, the signal intensity attenuation largely depends on the size of the absorber and it is expected that the stronger amplification will be needed for signals originating from small absorbers.

The very fast implementation of the proposed framework help in real-time simulation (and consequently aberration compensation) of PA waves in the entire human head in a PA computed tomography system.

In this study, we demonstrated that the forward mathematical modeling of the transmission effects (using deterministic ray-tracing principles) could explain most of the skull’s distorting effects. The results show that the skull-induced distortions on the PA signal and subsequently on the reconstructed images, are of paramount importance and indispensable. The focus of the current study was to develop a fast skull modeling method that in terms of accuracy is comparable to the state-of-the-art modeling algorithms, e.g., K-Wave. The fast implementation of the algorithm, ideally real-time, is required for practical purposes of skull aberration correction algorithm implementation and that was the main motivation of the proposed methodology. Considering non-smooth skull surface, i.e., integrating the results of this study in a finite-element model, will be the next step of this study. As another future work, we plan to use machine learning algorithms to train a kernel with the results of our simulations. Such kernel will then be used in real-time in the PA data processing unit for skull aberration correction and provide aberration corrected images. Various methods of learning could be used with all of them requiring large, realistic, and proven datasets for training then feeding those training data to the intelligent means of learning enabling it for real-time correction. In the meantime, we are considering artificial neural networks, vector space model and deep learning schemes to be used for this purpose. The real-time implementation of such framework for enhancement of transcranial PA images to compensate for the effects of skull aberrations, and therefore the clinical use of photoacoustic transcranial imaging, will be possible.

## 6. Conclusions

In this paper, we described the development of a fast modeling/simulation framework based on deterministic ray-tracing. The framework takes into account the frequency-dependent attenuation and dispersion effects that occur in wave reflection, refraction, and mode conversion at the skull surface. The framework allows mimicking 3-D full wave, linear and non-linear acoustic propagation in large media. In terms of running time, the proposed method significantly outperformed the k-Wave method when fine grid size is considered in the model. The four distorting effects including amplitude attenuation, phase distortion, multiple reflections, and mode conversion were then analyzed and confirmed by our method. The difference between results was less than 1.57% on average in numerical and analytical validation. We also studied these distorting effects on ex vivo skulls of mouse, rat, and dog and compared the results to those obtained from a similar simulation setup. The results were in a good agreement. Our simulations demonstrated that amplitude attenuation and phase distortion had the most significant distorting effect on the PA signal generated from the brain. Furthermore, skull thickness and target location relative to the inner-skull surface were the main parameters affecting PA signals distortion. The signal intensity attenuation largely depended on the size of the absorber. The key findings in this study using our simulator are as follows: the PA signal amplitude attenuation increased from 60% to 95.17% with increasing the skull thickness from 0.5 mm to 9.61 mm for a constant depth of target, i.e., 1.7 cm; The PA signal broadened from 1.86% to 63.17% and time shifted from 0.26 μs to 3.2 μs with the same change in skull thickness; the amplitude attenuation decreased from 38.24% to 11.39% and the signal broadening decreased from 24.71% to 4.80% with increasing the distance between target and the inner-skull surface from 1 cm to 3 cm at a constant thickness of skull, i.e., 7 mm. As a future work, we plan to develop a fast compensation algorithm based on artificial intelligence learning algorithms for a 3-D photoacoustic computed tomography imaging system.

## Figures and Tables

**Table 1:** Acoustic properties used in the simulations.

Symbol (Unit)	Soft Tissue	Skull	Water (Coupling Medium)
ρ (kg/m${}^{3}$)	1000 [39]	1800 [27]	1000 [65]
$c_{L}$ (m/s)	1500 [66]	2900 [27]	1486 [65]
$c_{S}$ (m/s)	—	1444 [27]	—
$\alpha_{0L}$ (Np/cm)	0.05 [66]	1.70 [39]	0.00 [65]
$y_{L}$	1.18 [66]	0.93 [39]	—
$\alpha_{0S}$ (Np/cm)	—	3.41 [39]	—
$y_{S}$	—	0.93 [39]	—

—: Data is not available.

**Table 2:** Normalized standard deviation (NSD) errors between the k-Wave and the proposed method for the simulation of different skull thicknesses with constant target depth, *d* = 1.7 cm.

Skull Thickness (mm)	NSD (%)
1	0.19
4	0.14
7	0.11

**Table 3:** Normalized standard deviation (NSD) errors between the k-Wave and the proposed method for the simulation of different target depths with a constant skull thickness, *h* = 7 mm.

Target Depth (cm)	NSD (%)
1.7	0.11
2.7	0.25
3.7	0.21

**Table 4:** Estimated errors ($E_{L}$ and $E_{S}$) between the analytical and numerical calculations of the longitudinal and shear intensity transmission coefficients, $I_{L}$ and $I_{S}$, for three different incidence angles.

$\theta_{i}$ (${}^{\circ }$)	$I_{L(\mathrm{anl})}$	$I_{S(\mathrm{anl})}$	$I_{L(\mathrm{num})}$	$I_{S(\mathrm{num})}$	$E_{L}$ (%)	$E_{S}$ (%)
0	0.414	0.000	0.421	0.000	1.69	0.00
15	0.192	0.026	0.185	0.025	−3.65	−3.85
30	0.133	0.048	0.139	0.046	4.51	−4.17

**Table 5:** Running time of k-Wave and the proposed method for different maximum frequency supported by grid size in k-Wave and two different number of frequencies modeled by our method.

$f_{\max}$ (MHz)	dx ${}^{a}$ (mm)	*N* ${}^{b}$	$t_{1}$ ${}^{c}$ (s)	$t_{2}$ ${}^{d}$ (s)	
				64 Frequency	32 Frequency
1	0.75	86${}^{3}$	849.45	35.06	19.74
1.5	0.5	128${}^{3}$	2571.89	40.20	20.16
3	0.25	256${}^{3}$	>18,000	42.66	22.14
5	0.15	427${}^{3}$	— ^e^	48.69	25.12
7.5	0.1	640${}^{3}$	— ^e^	59.14	27.92

${}^{a}$ dx: Grid size (applicable to k-Wave); ${}^{b}$*N*: Total number of elements (applicable to k-Wave); ${}^{c}$ $t_{1}$: k-Wave running time; ${}^{d}$ $t_{2}$: Our simulator running time; ^e^ —: Data is not available.

**Table 6:** Running time of k-Wave and the proposed method for the simulation of different skull thicknesses and two different target depths.

*h* (mm)	Target Depth = 1.7 cm				Target Depth = 3.7 cm			
	*N* ${}^{a}$	dx ${}^{b}$ (mm)	$t_{1}$ ${}^{c}$ (s)	$t_{2}$ ${}^{d}$ (s)	*N* ${}^{a}$	dx ${}^{b}$ (mm)	$t_{1}$ ${}^{c}$ (s)	$t_{2}$ ${}^{d}$ (s)
7	32${}^{3}$	1	20.21	149.18	64${}^{3}$	1	168.29	132.14
7.5	64${}^{3}$	0.5	168.88	149.83	128${}^{3}$	0.5	2533.10	135.21
7.25	128${}^{3}$	0.25	2535.67	147.26	256${}^{3}$	0.25	>18,000	134.63
7.1	320${}^{3}$	0.1	— ^e^	146.46	640${}^{3}$	0.1	— ^e^	133.16

${}^{a}$*N*: Total number of elements (applicable to k-Wave); ${}^{b}$ dx: Grid size (applicable to k-Wave); ${}^{c}$ $t_{1}$: k-Wave running time; ${}^{d}$ $t_{2}$: Our simulator running time; ^e^ —: Data is not available.

## References

[1] Wang L.V., Wu H.I. (2012). Biomedical Optics: Principles and Imaging.

[2] Wang L.V. (2009). Photoacoustic Imaging and Spectroscopy.

[3] Xu M., Wang L.V. (2006). Photoacoustic imaging in biomedicine. Rev. Sci. Instrum..

[4] Brecht H.P., Su R., Fronheiser M., Ermilov S.A., Conjusteau A., Oraevsky A.A. (2009). Whole-body three-dimensional optoacoustic tomography system for small animals. J. Biomed. Opt..

[5] Ntziachristos V., Razansky D. (2010). Molecular imaging by means of multispectral optoacoustic tomography (MSOT). Chem. Rev..

[6] Wang X., Pang Y., Ku G., Xie X., Stoica G., Wang L.V. (2003). Noninvasive laser-induced photoacoustic tomography for structural and functional in vivo imaging of the brain. Nat. Biotechnol..

[7] Burton N.C., Patel M., Morscher S., Driessen W.H., Claussen J., Beziere N., Jetzfellner T., Taruttis A., Razansky D., Bednar B. (2013). Multispectral opto-acoustic tomography (MSOT) of the brain and glioblastoma characterization. Neuroimage.

[8] Mohammadi-Nejad A.R., Mahmoudzadeh M., Hassanpour M.S., Wallois F., Muzik O., Papadelis C., Hansen A., Soltanian-Zadeh H., Gelovani J., Nasiriavanaki M. (2018). Neonatal brain resting-state functional connectivity imaging modalities. Photoacoustics.

[9] Meimani N., Abani N., Gelovani J., Avanaki M.R. (2017). A numerical analysis of a semi-dry coupling configuration in photoacoustic computed tomography for infant brain imaging. Photoacoustics.

[10] Kang J., Boctor E.M., Adams S., Kulikowicz E., Zhang H.K., Koehler R.C., Graham E.M. (2018). Validation of Noninvasive Photoacoustic Measurements of Sagittal Sinus Oxyhemoglobin Saturation in Hypoxic Neonatal Piglets. J. Appl. Physiol..

[11] Kang J., Zhang H., Rahmim A., Wong D., Kang J., Boctor E. Toward high-speed transcranial photoacoustic imaging using compact near-infrared pulsed LED illumination system. Proceedings of the Photons Plus Ultrasound: Imaging and Sensing 2017.

[12] Nie L., Guo Z., Wang L.V. (2011). Photoacoustic tomography of monkey brain using virtual point ultrasonic transducers. J. Biomed. Opt..

[13] Yang X., Wang L.V. (2008). Monkey brain cortex imaging by photoacoustic tomography. J. Biomed. Opt..

[14] Yao J., Xia J., Maslov K.I., Nasiriavanaki M., Tsytsarev V., Demchenko A.V., Wang L.V. (2013). Noninvasive photoacoustic computed tomography of mouse brain metabolism in vivo. Neuroimage.

[15] Li L., Xia J., Li G., Garcia-Uribe A., Sheng Q., Anastasio M.A., Wang L.V. (2016). Label-free photoacoustic tomography of whole mouse brain structures ex vivo. Neurophotonics.

[16] Yao J., Wang L., Yang J.M., Maslov K.I., Wong T.T., Li L., Huang C.H., Zou J., Wang L.V. (2015). High-speed label-free functional photoacoustic microscopy of mouse brain in action. Nat. Methods.

[17] Lin L., Xia J., Wong T.T., Li L., Wang L.V. (2015). In vivo deep brain imaging of rats using oral-cavity illuminated photoacoustic computed tomography. J. Biomed. Opt..

[18] Li M.L., Oh J.T., Xie X., Ku G., Wang W., Li C., Lungu G., Stoica G., Wang L.V. (2008). Simultaneous molecular and hypoxia imaging of brain tumors in vivo using spectroscopic photoacoustic tomography. Proc. IEEE.

[19] Hu S., Maslov K., Tsytsarev V., Wang L.V. (2009). Functional transcranial brain imaging by optical-resolution photoacoustic microscopy. J. Biomed. Opt..

[20] Nasiriavanaki M., Xia J., Wan H., Bauer A.Q., Culver J.P., Wang L.V. (2014). High-resolution photoacoustic tomography of resting-state functional connectivity in the mouse brain. Proc. Natl. Acad. Sci. USA.

[21] Li M., Tang Y., Yao J. (2018). Photoacoustic tomography of blood oxygenation: A mini review. Photoacoustics.

[22] Zhang P., Li L., Lin L., Hu P., Shi J., He Y., Zhu L., Zhou Y., Wang L.V. (2018). High-resolution deep functional imaging of the whole mouse brain by photoacoustic computed tomography in vivo. J. Biophotonics.

[23] Gottschalk S., Felix Fehm T., Luís Deán-Ben X., Razansky D. (2015). Noninvasive real-time visualization of multiple cerebral hemodynamic parameters in whole mouse brains using five-dimensional optoacoustic tomography. J. Cereb. Blood Flow Metab..

[24] Gottschalk S., Fehm T.F., Deán-Ben X.L., Tsytsarev V., Razansky D. (2016). Correlation between volumetric oxygenation responses and electrophysiology identifies deep thalamocortical activity during epileptic seizures. Neurophotonics.

[25] Balasundaram G., Ding L., Li X., Attia A.B.E., Dean-Ben X.L., Ho C.J.H., Chandrasekharan P., Tay H.C., Lim H.Q., Ong C.B. (2018). Noninvasive Anatomical and Functional Imaging of Orthotopic Glioblastoma Development and Therapy using Multispectral Optoacoustic Tomography. Transl. Oncol..

[26] Wang T., Jing Y. (2015). A fast marching method based back projection algorithm for photoacoustic tomography in heterogeneous media. arXiv.

[27] Kneipp M., Turner J., Estrada H., Rebling J., Shoham S., Razansky D. (2016). Effects of the murine skull in optoacoustic brain microscopy. J. Biophotonics.

[28] Estrada H., Rebling J., Turner J., Razansky D. (2016). Broadband acoustic properties of a murine skull. Phys. Med. Biol..

[29] Hynynen K., Jolesz F.A. (1998). Demonstration of potential noninvasive ultrasound brain therapy through an intact skull. Ultrasound Med. Biol..

[30] Hynynen K., Sun J. (1999). Trans-skull ultrasound therapy: The feasibility of using image-derived skull thickness information to correct the phase distortion. IEEE Trans. Ultrasonics Ferroelectr. Freq. Control.

[31] Clement G., Hynynen K. (2002). A non-invasive method for focusing ultrasound through the human skull. Phys. Med. Biol..

[32] Aubry J.F., Tanter M., Pernot M., Thomas J.L., Fink M. (2003). Experimental demonstration of noninvasive transskull adaptive focusing based on prior computed tomography scans. J. Acoust. Soc. Am..

[33] Marquet F., Pernot M., Aubry J., Montaldo G., Marsac L., Tanter M., Fink M. (2009). Non-invasive transcranial ultrasound therapy based on a 3D CT scan: protocol validation and in vitro results. Phys. Med. Biol..

[34] Pinton G., Aubry J.F., Fink M., Tanter M. (2011). Effects of nonlinear ultrasound propagation on high intensity brain therapy. Med. Phys..

[35] Jones R.M., O’Reilly M.A., Hynynen K. (2013). Transcranial passive acoustic mapping with hemispherical sparse arrays using CT-based skull-specific aberration corrections: A simulation study. Phys. Med. Biol..

[36] Pinton G., Aubry J.F., Bossy E., Muller M., Pernot M., Tanter M. (2012). Attenuation, scattering, and absorption of ultrasound in the skull bone. Med. Phys..

[37] Treeby B.E., Cox B. (2010). Modeling power law absorption and dispersion for acoustic propagation using the fractional Laplacian. J. Acoust. Soc. Am..

[38] Jin X., Li C., Wang L.V. (2008). Effects of acoustic heterogeneities on transcranial brain imaging with microwave-induced thermoacoustic tomography. Med. Phys..

[39] Schoonover R.W., Wang L.V., Anastasio M.A. (2012). Numerical investigation of the effects of shear waves in transcranial photoacoustic tomography with a planar geometry. J. Biomed. Opt..

[40] Fry F., Barger J. (1978). Acoustical properties of the human skull. J. Acoust. Soc. Am..

[41] Treeby B.E. (2013). Acoustic attenuation compensation in photoacoustic tomography using time-variant filtering. J. Biomed. Opt..

[42] Deán-Ben X.L., Razansky D., Ntziachristos V. (2011). The effects of acoustic attenuation in optoacoustic signals. Phys. Med. Biol..

[43] Roitner H., Burgholzer P. (2010). Efficient modeling and compensation of ultrasound attenuation losses in photoacoustic imaging. Inverse Probl..

[44] Treeby B., Pan J. (2009). A practical examination of the errors arising in the direct collocation boundary element method for acoustic scattering. Eng. Anal. Bound. Elem..

[45] Mohammadi L., Behnam H., Tavakkoli J., Nasiriavanaki M. (2018). Skull’s acoustic attenuation and dispersion modeling on photoacoustic signal. Photons Plus Ultrasound: Imaging and Sensing 2018.

[46] Huang C., Nie L., Schoonover R.W., Guo Z., Schirra C.O., Anastasio M.A., Wang L.V. (2012). Aberration correction for transcranial photoacoustic tomography of primates employing adjunct image data. J. Biomed. Opt..

[47] Huang C., Nie L., Schoonover R.W., Wang L.V., Anastasio M.A. (2012). Photoacoustic computed tomography correcting for heterogeneity and attenuation. J. Biomed. Opt..

[48] Schoonover R.W., Anastasio M.A. (2011). Compensation of shear waves in photoacoustic tomography with layered acoustic media. JOSA A.

[49] Estrada H., Huang X., Rebling J., Zwack M., Gottschalk S., Razansky D. (2018). Virtual craniotomy for high-resolution optoacoustic brain microscopy. Sci. Rep..

[50] Kyriakou A., Neufeld E., Werner B., Székely G., Kuster N. (2015). Full-wave acoustic and thermal modeling of transcranial ultrasound propagation and investigation of skull-induced aberration correction techniques: A feasibility study. J. Ther. Ultrasound.

[51] Odabaee M., Odabaee M., Pelekanos M., Leinenga G., Götz J. (2018). Modeling ultrasound propagation through material of increasing geometrical complexity. Ultrasonics.

[52] Nagatani Y., Imaizumi H., Fukuda T., Matsukawa M., Watanabe Y., Otani T. (2006). Applicability of finite-difference time-domain method to simulation of wave propagation in cancellous bone. Jpn. J. Appl. Phys..

[53] Yoon K., Lee W., Croce P., Cammalleri A., Yoo S.S. (2018). Multi-resolution simulation of focused ultrasound propagation through ovine skull from a single-element transducer. Phys. Med. Biol..

[54] Pernot M., Aubry J.F., Tanter M., Thomas J.L., Fink M. Experimental validation of 3D finite differences simulations of ultrasonic wave propagation through the skull. Proceedings of the 2001 IEEE Ultrasonics Symposium.

[55] Treeby B.E., Cox B.T. (2010). k-Wave: MATLAB toolbox for the simulation and reconstruction of photoacoustic wave fields. J. Biomed. Opt..

[56] Jocker J. (2005). Ultrasonic Wave Propagation in Heterogeneous Elastic And Poroelastic Media.

[57] Firouzi K., Saffari N. (2015). A numerical model for the study of photoacoustic imaging of brain tumours. arXiv.

[58] Guo X., Zhang D., Gong X. (2006). Evaluation of ultrasonic scattering in human cancellous bone by using a binary mixture model. Phys. Med. Biol..

[59] Szabo T.L. (2004). Diagnostic Ultrasound Imaging: Inside Out.

[60] Waters K.R., Hughes M.S., Mobley J., Brandenburger G.H., Miller J.G. (2000). On the applicability of Kramers–Krönig relations for ultrasonic attenuation obeying a frequency power law. J. Acoust. Soc. Am..

[61] Waters K.R., Mobley J., Miller J.G. (2005). Causality-imposed (Kramers-Kronig) relationships between attenuation and dispersion. IEEE Trans. Ultrason. Ferroelectr. Freq. Control.

[62] Szabo T.L. (1994). Time domain wave equations for lossy media obeying a frequency power law. J. Acoust. Soc. Am..

[63] Chen W., Holm S. (2003). Modified Szabo’s wave equation models for lossy media obeying frequency power law. J. Acoust. Soc. Am..

[64] White P., Clement G., Hynynen K. (2006). Longitudinal and shear mode ultrasound propagation in human skull bone. Ultrasound Med. Biol..

[65] Clement G., White P., Hynynen K. (2004). Enhanced ultrasound transmission through the human skull using shear mode conversion. J. Acoust. Soc. Am..

[66] Nam K., Rosado-Mendez I.M., Rubert N.C., Madsen E.L., Zagzebski J.A., Hall T.J. (2011). Ultrasound attenuation measurements using a reference phantom with sound speed mismatch. Ultrason. Imaging.

[67] Hoelen C., De Mul F. (1999). A new theoretical approach to photoacoustic signal generation. J. Acoust. Soc. Am..

[68] Hayner M., Hynynen K. (2001). Numerical analysis of ultrasonic transmission and absorption of oblique plane waves through the human skull. J. Acoust. Soc. Am..

[69] Boashash B. (2015). Time-Frequency Signal Analysis and Processing: A Comprehensive Reference.

[70] De Greve B. (2004). Reflections and Refractions in Ray Tracing.

[71] Mozaffarzadeh M., Mahloojifar A., Orooji M., Kratkiewicz K., Adabi S., Nasiriavanaki M. (2018). Linear-array photoacoustic imaging using minimum variance-based delay multiply and sum adaptive beamforming algorithm. J. Biomed. Opt..

[72] Mozaffarzadeh M., Mahloojifar A., Orooji M., Adabi S., Nasiriavanaki M. (2018). Double-stage delay multiply and sum beamforming algorithm: Application to linear-array photoacoustic imaging. IEEE Trans. Biomed. Eng..

[73] Hariri A., Fatima A., Mohammadian N., Mahmoodkalayeh S., Ansari M.A., Bely N., Avanaki M.R. (2017). Development of low-cost photoacoustic imaging systems using very low-energy pulsed laser diodes. J. Biomed. Opt..

[74] Wang X., Chamberland D.L., Xi G. (2008). Noninvasive reflection mode photoacoustic imaging through infant skull toward imaging of neonatal brains. J. Neurosci. Methods.

[75] Ruan J., Prasad P. (2001). The effects of skull thickness variations on human head dynamic impact responses. Stapp. Car Crash J..

